# Antituberculosis Drug Nonadherence and Its Associated Factors: Evidence from Debre Berhan Town, North Shewa Zone, Ethiopia

**DOI:** 10.1155/2023/7645058

**Published:** 2023-04-29

**Authors:** Tomas Getahun, Hailemikael Debebe, Helen Getahun, Yonas Abebe, Kidist Assefa, Mizan Habtemichael

**Affiliations:** ^1^Armauer Hansen Research Institute-Ministry of Health, Ethiopia; ^2^Debre Berhan University, College of Health Sciences, Department of Nursing, Ethiopia; ^3^School of Public Health, College of Health Sciences, Addis Ababa University, Addis Ababa, Ethiopia; ^4^Gandhi Memorial Hospital-Addis Ababa Regional Bureau, Addis Ababa, Ethiopia

## Abstract

**Introduction:**

Tuberculosis is a bacterial disease caused by the *Mycobacterium tuberculosis*. Regardless of many efforts made to control tuberculosis, the disease remains to be a major public health problem. Nonadherence to antituberculosis treatment poses a challenge to the disease treatment as it potentially increases the risk of drug resistance, mortality, relapse, and extended infectiousness. The North Shewa Zone had a poor performed on TB control status, so this study assessed the prevalence of antituberculosis drug nonadherence and its associated factors at governmental health institutions in Debre Berhan town, North Shewa Zone, Ethiopia, 2020.

**Methods:**

An institution-based cross-sectional study design was employed. A total of 180 tuberculosis patients were included in the study. The data was entered using EpiData version 3.1 and exported to SPSS version 20.0 for statistical analysis. Bivariable and multivariable logistic regression analyses were computed to determine factor associated with antituberculosis drug nonadherence.

**Result:**

Study finding shows that 26.0% respondents were nonadherent to their antituberculosis treatment. Respondents who were married were less likely to be nonadherent than who were single (AOR = 0.307; 95%CI = 0.120, 0.788). Respondents who have primary and secondary education were less likely to be nonadherent than those who had no formal education (AOR = 0.313; 95%CI = 0.100, 0.976). Respondents who experienced drug side effects were two times more likely to be nonadherent than those who did not experience drug side effects (AOR = 2.379; 95%CI = 1.008, 5.615). In addition, respondents who do not screen for HIV were four times more likely to be nonadherent than their counterparts (AOR = 4.620; 95%CI = 11.135, 18.802).

**Conclusion:**

The antituberculosis drug nonadherence is high. Marital status, educational status, drug side effects, HIV screening status of the patients, and availability of medication were the variables that influence drug nonadherence. There is a need to strengthen awareness creation and improve quality of the TB treatment services and anti-TB drug availability.

## 1. Introduction

Tuberculosis (TB) is a bacterial infectious disease caused by the *Mycobacterium tuberculosis*. Even though the disease is preventable and curable, it is among the top killer infectious diseases worldwide [1]. Having a compromised immune system due to different disease conditions, HIV/AIDS, diabetes mellitus, malnutrition, and chronic renal failure are some of the risk factors for developing TB. [2]. Every year, 1.5 million people die from TB and 10 million people fall ill. Even though TB is found around the world, 95% morbidity and 99% mortality because of TB occur in low- and middle-income countries. It is also the leading cause of death of people with HIV and a major contributor to antimicrobial resistance [1, 3].

Africa region covers about a quarter of the TB burden, and it has the uppermost TB morbidity burden per capita [4, 5]. Regarding TB incidence, Ethiopia is among the top 16 countries worldwide and one of the top three in Africa region. In Ethiopia, over one-third of the total population has been exposed to the TB. In 2018, 377,030 Ethiopians have active TB of all forms, with more than 165,000 new cases [6, 7].

Directly observed treatment (DOT) is among the extensively applied and elongated intervention worldwide in health sector. In Ethiopia, a control approach for TB was started in the early 1960s with the formation of TB centers in a limited areas of the country, which then followed by the DOT package in the 1990s [8].

According to WHO standard treatment guideline for active TB, the treatment is completed in two phases where the initial phase is composed of four drugs and a continuation phase has two drugs. The regimen symbolizations show the number of months for which a relevant combination of medicines and dosage is used and if certain drugs are used for a different duration. For example, 2HREZ/4HR3 indicates isoniazid, rifampicin, ethambutol, and pyrazinamide daily for two months, followed by four months of isoniazid and rifampicin given three times a week [9].

In spite of the application of globally suggested DOT approach, the WHO revealed that a significant number of TB cases are unsuccessful after numerous treatments; many of the patients relapsed after completion of their treatment, several had to undertake retreatment after finishing their treatment, and most of the patients developed multi-drug-resistant TB (MDR-TB) [10].

Evidences showed that treatment nonadherence is the main reason for poor TB treatment outcomes, occurrence of MDR-TB, and extended infectiousness [11, 12]. Worldwide studies have found that treatment nonadherence is a concern for the public. For instance, according to a health facility-based cross-sectional study conducted in Pakistan, 34.5% of the study participants reported nonadherence to anti-TB drug [13]. Similarly, another cross-sectional study conducted in Turkey found that 34.5% patient met the criteria of nonadherence [14].

Another cross-sectional study conducted in Tanzania showed that out of the 617 patients, 8% were nonadherent for TB treatment [15].

In Ethiopia, even though TB treatments are given free of charge, TB continues to be a main health problem and leading cause of mortality. The national program for TB prevention and control praises DOT as the main approach for disease control, but its application is poor in different areas of the country [8]. In a prospective cross-sectional research done in Jimma Hospital, about 12% of patients were nonadherent for anti-TB medication [16]. In similar institutional-based cross-sectional studies conducted at Arba Minch and Gonder town, Ethiopia, the treatment of TB nonadherence was 24.7% and 21.2%, respectively [12, 17].

The Amhara region, North Shewa Zone particularly, had a poor performance on TB control activities. Yet, as far as the investigators' knowledge, there is no research done on the magnitude of TB treatment nonadherence in this study area. Therefore, the aim of the current study is identifying the magnitude of nonadherence for TB treatment and possible associated factors. The results may contribute for policy makers and health departments to produce a plan for better treatment outcome, reduce recurrence of tuberculosis, and develop multi-drug-resistant TB. It can also serve as input for further study on this area.

## 2. Methods and Materials

### 2.1. Study Setting and Period

This study was conducted in Debre Berhan town, which is the capital city of North Shewa Zone, found at 130 km in the North East direction from Addis Ababa. It has a total of 97,969 populations where 53,883 are female and 44,086 are male. Administratively, Debre Berhan town is subdivided into nine kebeles (public administration units). In the town, there is one governmental referral hospital, one private general hospital, three health centers, and 12 private clinics. There are also 9 urban and 5 rural health posts. Among them, one government referral hospital, one private hospital, three health centers, and one clinic in Debre Berhan prison provide TB diagnosis and treatment service.

### 2.2. Study Design and Period

A health facility-based cross-sectional study was conducted from February 1 up to February 30, 2020.

### 2.3. Eligibility Criteria

All TB patients aged 18 years and above and who were on anti-TB treatment for at least 1 month at the time of the study were included in the study. Those TB patients who are seriously ill and unable to communicate were excluded from the study.

### 2.4. Sample Size and Sampling Technique

The total sample size for the study was determined by using a single population proportion formula by taking proportion of anti-TB drug adherence in southwest Ethiopia 88% [16], 95% confidence level 5% margin of error, and 10% nonresponse rate. Then, the final sample size became 180.

This study was done on 4 government health facilities who are providing TB diagnosis and treatment: Debre Berhan Referral Hospital, Debre Berhan Health Center, Ayer Tena Health Center, and Tebasie Health Center. The required sample size was proportionally allocated to the 4 governmental health facilities in the town based on the number of patients on anti-TB treatment. Based on the patient register of each facility, systematic random sampling was employed to select respondents.

### 2.5. Study Variables and Measurement

Our main outcome variable (dependent variable) was nonadherence to anti-TB treatment. Sociodemographic factors, TB therapy, disease-related factors, patient-related factors, behavioral factor, and health system-related factors were independent variables.

### 2.6. Operational Definitions

Adherence to anti-Tb drug: “The extent to which patients' history of therapeutics drug taking coincides with prescribed treatment”

- Adherent: “Patients who take their medication missing no dose during their treatment period”
- Nonadherent: “Patients who missed their medication schedule at least once during their treatment period”

Knowledge about Tb and its treatment: patients' understanding about TB transmission, diagnosis, and treatment

- Good knowledge: total knowledge score above the median value
- Poor knowledge: total knowledge score below the median value

### 2.7. Data Collection Tool and Procedure

A structured questionnaire containing both open- and close-ended questions was used to determine participant's anti-TB drug nonadherence and its associated factors. It is developed based on the objectives of the study. The questioner contains questions on patient's adherence practice for the anti-TB treatment, sociodemographic and economic characters, health-related factors, behavioral factor, and health system-related factors. Four data collectors (clinical nurses) working at the DOT clinic and one supervisor were recruited. They were trained for two days on how to collect data by using questionnaire and general information about the contents of the data collection tool. Data was collected with close supervision by principal investigators and supervisor.

### 2.8. Data Quality Assurance

The questionnaire was first prepared in English language, and then, it is translated to Amharic language by professional translators. To check the uniformity of responses and understandability of the questions, the questionnaire was pretested. After the pretest, the possible modification was made on the tool. The principal investigator and supervisor were daily supervising the data collection process, and they checked for completeness and uniformity of responses given at the end of each day.

### 2.9. Data Processing and Analysis

Data was entered and cleaned using EpiData version 3.3 and exported into SPSS version 20.0 for analysis. Frequency tables, graphs, and descriptive summaries were used to describe the study variables. Bivariate logistic regression analysis was used to see the significance of the association between anti-TB treatment nonadherence and independent variables. In the bivariate analysis, variables with *p* value ˂ 0.20 were transferred to the multivariate analysis in order to control confounders. Odds ratio with 95% CI was computed to measure the strength of the association between the outcome and the explanatory variables. *p* value ˂ 0.05 was considered as statistically significant.

## 3. Result

### 3.1. Sociodemographic Characteristics

Out of the identified 192 respondents for the study, 180 of them participated by making the 93.8% response rate. The majority, 127 (70.6%), of respondents were rural residents. Around one hundred three (57.2%) of respondents were male, and 74 (41.1%) were in the age group of 20 to 29 years with the median age of 30 years. About half (48.9%) of respondents were married, and 44 (52.2%) had primary and secondary educational status. Regarding monthly income, 69 (38.3%) of them earned 1000 to 2000 birr per month (Table 1).

### 3.2. Knowledge Level about TB and Its Treatment

Regarding respondent knowledge about TB, 174 (96.7%) of them have heard about the diseases, and their source of information, for 116 (64.4%) of them, were health professionals. Majority, 161 (89.4%), of respondents reported that TB is transmitted by air. For the questions asked to assess respondents' knowledge on sign and symptom of TB, about half (51.1%) of respondents mentioned that vomiting is a symptom for TB. About 127 (70.6%) of them know that there are TB diagnostic tools.

One hundred twenty-seven (70.6%) respondents said that the disease can affect both rich and poor groups. Most of the respondents, 115 (63.9%), thought that the treatment of TB lasts from six to eight months, and 168 (93.3%) of them understood that TB can be cured (Table 2). The overall composite knowledge score was calculated by adding all items measuring knowledge, showing that 24.4% of them had good knowledge level and 75.6% of respondents had poor knowledge level.

### 3.3. Health Status and Behavioral Characteristics

Around 133 (73.9%) of respondents had smear positive for PTB, about 92 (51.1%) of respondents experienced side effects from the anti-TB therapy, and diarrhea and vomiting were experienced by 47 (51.0%). Around 84.2% of them were taking the drug with the monitor of their spouse, parents, and siblings (Table 3).

Of the total, 165 (91.7%) respondents were screened for HIV and 27 (16.3%) were positive. One hundred sixty-five (91.7%) respondents were taking medications other than anti-TB treatment; among them, 22 (13.33%) were taking highly active antiretroviral therapy (HAART) (Table 4). Among the TB patients, 4.4% were cigarette smokers, 21.7% consumes alcohol, and 8.3% were khat chewers.

### 3.4. Healthcare System and Other Related Characteristics

Concerning the healthcare system, more than half (56.7%) of respondents said that it only took them less than 30 minutes to reach to the health institution, and 126 (70.0) of them paid over 50 birr for transportation. Majority, 159 (88.3%), of respondents said that the waiting time to get services was less than thirty minutes, and 174 (96.7%) of them replied that drugs were always available in the health institution (Table 5).

### 3.5. Anti-TB Treatment Nonadherence Status

Most respondents, 161 (89.4%), were on 2HERZ/4HR or 2HERZ/6HE anti-TB regimen. About one-third (36.7%) of respondents have been taking the drugs for one to two months. About 114 (63.3%) of them swallow three tablets per day (Table 3).

**Figure 1:**
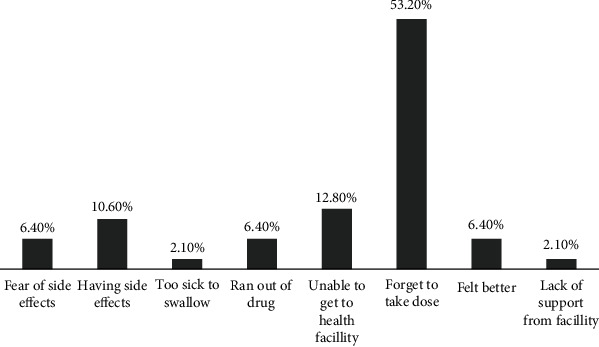
Reasons for nonadherence to TB patients, Debre Berhan health facilities, Ethiopia, 2020.

Nearly one-fourth (26.0%) of respondents were nonadherent to their anti-TB treatment. Forgetting to take the drugs was mentioned by 53.2% of respondents as a reason for missing medication. About 12.8% of respondents mentioned that their reason for a missed dose was inability to go to a health facility. A few (2.1%) mentioned that lack of support caused the nonadherence from facility while 10.6% of respondents stated that having side effects was hindering them from adhering (Figure 1).

### 3.6. Factors Associated with Anti-TB Drug Nonadherence

In bivariable logistic regression analysis, variables with *p* value < 0.2 were included during multiple logistic regression analysis (Table 6). In multivariable logistic regression analysis, marital status, educational level, side effects of drug, screening status for HIV, and availability of medicine revealed a significant association with anti-TB drug nonadherence. Respondents who were married were less likely (AOR = 0.307; 95%CI = 0.120, 0.788) to be nonadherent than respondents who were single. Respondents who have primary and secondary education were less likely (AOR = 0.313; 95%CI = 0.100, 0.976) than those who had no formal education. Respondents who experienced drug side effects were two times (AOR = 2.379; 95%CI = 1.008, 5.615) more likely to be nonadherent than those who did not experience drug side effects. In addition, respondents who do not screen for HIV were four times (AOR = 4.620; 95%CI = 11.135, 18.802) more likely to be nonadherent than their counterparts. Respondents who reported medication sometimes available were three times (AOR = 3.845; 95%CI = 1.288, 8.783) more likely to be nonadherent than those who said medication is always available.

## 4. Discussion

The main purpose of this study was to assess the level of anti-TB drug nonadherence and its associated factor among TB patient attending health institution in Debre Berhan town. Even though full anti-TB medication adherence is expected in accordance to DOT strategy, the study finding revealed that 26.0% of TB patient did not adhere to their anti-TB treatment [8].

This finding is in line with the study conducted in southeast Nigeria that reported 24.2% of nonadherence among TB patients attending tertiary level health institutions. Similar finding reported from study conducted in southern Ethiopia, which examined anti - TB drug non-adherence among TB patients, the non-adherence rate was 24.7%. The proportion of nonadherence found in this study is higher than the result of a cross-sectional study on TB patient receiving DOT in Mumbai, India, where only 16.0% of respondents were nonadherent [18]. This discrepancy can be because of the difference in the study population.

In the current study, the main reason for nonadherence was forgetting to take medication, inability to go to health institution, and fear of drug side effects. Similar finding was revealed from study conducted in Hadiya Zone, southern Ethiopia. Fear of side effect was also one reason for nonadherence to anti-TB medication from a study done in Tigray, Northern Ethiopia [19]. In our finding, 51.1% of respondents mentioned vomiting as one symptom of TB which probably indicates that there is a gap in clearly differentiating between sign and symptoms of the disease itself and side effect of anti-TB medications. Fear of drug side effect is indicated among the main reasons for nonadherence, and the possible reason for this could be lack of knowledge on the expected side effects of the anti-TB therapy and the TB itself.

In the current study, one of the factors that showed significant association with anti-TB drug adherence was respondents' marital status; TB patients who were married were more likely to adhere to their treatment. In contrast, a study conducted in Arba Minch, Southern Ethiopia, revealed that marital status has no effect on TB patient's drug nonadherence; this discrepancy could be because of the difference in the background characteristics of the study population [17].

In this study, respondent educational status was identified as one factor for anti-TB medication nonadherence. This study found that patients who had primary and secondary educational status were less likely to be nonadherent. This finding is in line with the study conducted in southwest Ethiopia and southeast Nigeria in which respondent educational level of TB patients has a statistically significant association with anti-TB drug adherence. This might be because those patients who were less educated have a less understanding about the treatment failure and disease relapse [20].

## 5. Conclusion

This study found that TB patient's anti-TB drug nonadherence in Debre Berhan town health institution is high. The main reason of anti-TB drug nonadherence was forgetting to take dose and inability to go to health facility, marital status, educational level, drug side effect, history of HIV screening, and availability of the drug influences treatment nonadherence.

There should be strong monitoring and evaluation of anti-TB treatment service provision, and it should be improved in relation to availability of drugs and developing defaulter tracing mechanism. Woreda Health Office and other primary healthcare unit should strengthen information dissemination and health education on possible drug side effects at all phase of treatment.

## Figures and Tables

**Table 1:** Sociodemographic characteristics of respondents, Debre Berhan health facilities, Ethiopia, 2020 (*n* = 180).

Characteristics	Frequency (*n* = 180)	Percent (%)
Age		
<20	13	7.2
20 to 29	74	41.1
30 to 39	44	24.4
40 >	49	27.2
Sex		
Male	103	57.2
Female	77	42.8
Place of residence		
Urban	53	29.4
Rural	127	70.6
Marital status		
Single	88	48.9
Married	68	37.8
Divorced	13	7.2
Windowed	11	6.1
Educational level		
No formal education	33	18.3
Primary and secondary	94	52.2
College diploma and above	53	29.4
Monthly income		
Less than 1000 birr	47	26.1
1000 to 2000 birr	69	38.3
Above 2000 birr	64	35.6
Occupation		
Farmer	70	38.9
Government employee	46	25.6
Private employee	38	21.1
Merchant	13	7.2
Housewife	13	7.2

**Table 2:** Knowledge about TB and its treatment of respondents, Debre Berhan health facilities, Ethiopia, 2020 (*n* = 180).

Characteristics	Frequency	Percent (%)
TB mode of transmission		
Air	161	89.4
Food	1	0.6
Do not know	17	9.4
Others	1	0.6
Sign and symptom of TB		
Cough >2 weeks	24	13.3
Loss of weight	3	1.7
General weakness	9	5.0
Vomiting	92	51.1
Do not know	52	28.9
Is there diagnostic tool for TB?		
Yes	127	70.6
No	3	1.7
Do not know	50	27.8
Socioeconomic status mostly affected by TB		
Poor	43	23.9
Rich	1	0.6
Both groups	121	67.2
Do not know	15	8.3
How long is the treatment for TB?		
6 to 8 months	115	63.9
8 to 12 months	22	12.2
Do not know	43	23.9
Can TB be cured?		
Yes	168	93.3
No	12	6.7

**Table 3:** Anti-TB therapy-related characteristics of TB patients, Debre Berhan health facilities, Ethiopia, 2020 (*n* = 180).

Characteristics	Frequency	Percent (%)
Type of TB		
PTB +ve	133	73.9
PTB –ve	29	16.1
EPTB	9	5.0
Do not know	9	5.0
TB regimen		
2HERZ/4HR or 2HERZ/6HE	161	89.4
2HERZ/4HR	17	9.4
Others	2	1.2
How long you take anti-TB drugs		
1 to 2 months	66	36.7
3 to 4 months	59	32.8
Greater than 4 months	55	30.6
How many tablets you swallow per day		
One	3	1.7
Two	10	5.6
Three	114	63.3
Four	53	29.4
Does anyone monitor you with drug taking?		
Yes	152	84.2
No	28	15.6
If yes, who?		
Spouse	48	31.4
Father	7	4.6
Mother	48	31.4
Sibling	36	23.5
Friend	13	9.1
Experience side effects		
Yes	92	51.1
No	88	48.9
Type of side effects		
Diarrhea and vomiting	47	26.1
Headache and dizziness	27	15.0
Skin rash	8	4.4
Numbness on feet or hand	10	5.6
How long medicine taken before felt better		
Less than 2 months	48	26.7
2 to 4 months	115	63.9
5 to 6 months	14	7.8
Did not feel better	3	1.7

**Table 4:** Health-related characteristics of TB patients at Debre Berhan health facilities, Ethiopia, 2020 (*n* = 180).

Characteristics	Frequency	Percent (%)
Screened for HIV		
Yes	165	91.7
No	15	8.3
HIV status		
Positive	27	16.3
Negative	137	83.03
Not known	16	9.69
Do you take medication besides anti-TB treatment?		
Yes	148	82.2
No	32	17.8
What type of medication you take beside anti-TB		
HAART	22	13.33
Psychiatric	4	2.2
Antihypertensive	5	2.8
Others	117	65.0

**Table 5:** Healthcare system-related characteristics of TB patients, Debre Berhan health facilities, Ethiopia, 2020 (*n* = 180).

Healthcare system-related characteristics	Frequency	Percent (%)
Time take to reach health institution		
Less than 30 minutes	102	56.7
30 to 60 minutes	74	41.1
Greater than 60 minutes	4	2.2
How much paid for transportation		
Did not pay	54	30.0
Greater than 50 birr	126	70.0
How long you wait in a health institution		
Less than 30 minutes	159	88.3
Greater than 30 minutes	21	11.7
Availability of medicine		
Always available	174	96.7
Sometimes available	6	3.3

**Table 6:** Logistic regression analysis of factors associated with anti-TB treatment adherence, Debre Berhan health facilities, 2020.

Characteristics	Nonadherence		COR (95% CI)	AOR (95% CI)
	Yes	No		
Marital status				
Single	25	63	1	1
Married	12	56	0.540 (0.248, 1.174)	0.307 (0.120, 0.788)^∗^
Divorced	5	8	1.575 (0.470, 5.280)	1.905 (0.442, 8.210)
Windowed	5	6	0.254 (0.587, 7.508)	0.950 (0.161, 5.614)
Educational level				
No formal education	14	19	1	1
Primary and secondary	14	80	0.238 (0.097, 0.581)^∗^	0.313 (0.100, 0.976)^∗^
College diploma and above	19	34	0.758 (0.321, 1.846)	1.039 (0.301, 3.583)
Experience side effects				
Yes	34	56	3.596 (1.740, 7.432)^∗^	2.379 (1.008, 5.615)^∗^
No	13	77	1	1
Screened for HIV				
Yes	38	127	1	1
No	9	6	5.01 (1.678, 14.981)^∗^	4.620 (1.135, 18.802)^∗^
How much paid for transportation				
Did not pay	9	45	1	1
Greater than 50 birr	38	88	2.159 (0.960, 4.856)	1.667 (0.629, 4.416)
Availability of medicine				
Always available	42	132	1	1
Sometimes available	5	1	15.74 (1.785, 23.833)	3.845 (1.288, 8.783)^∗^
Do you smoke cigarettes?				
Yes	4	4	3.000 (0.719, 12.514)	1.508 (0.279, 8.146)
No	43	129	1	1
Do you drink alcohol?				
Yes	15	240	1	1
No	32	109	2.129 (1.000, 4.534)^∗^	1.468 (0.544, 3.963)

^∗^Significantly associated.

## Data Availability

The corresponding author will provide the dataset utilized for this study upon rational request.

## Abbreviations

AIDS: Acquired immune deficiency syndrome
ART: Antiretroviral therapy
DBRH: Debre Berhan Referral Hospital
DOTS: Directly observed treatment of short course
EPTB: Extra pulmonary tuberculosis
HC: Health center
HF: Health facility
HIV: Human immunodeficiency virus
MDR-TB: Multi-drug-resistant tuberculosis
MTB: *Mycobacterium tuberculosis*
OR: Odds ratio
PTB: Pulmonary tuberculosis
PTB-SM+: Pulmonary tuberculosis smear positive
PTB-SM-: Pulmonary tuberculosis smear negative
SPSS: Statistical Package for the Social Sciences
TB: Tuberculosis
WHO: World Health Organization.
